# Genetic analysis of the invasive alga *Didymosphenia geminata* in Southern Argentina: Evidence of a Pleistocene origin of local lineages

**DOI:** 10.1038/s41598-019-55155-1

**Published:** 2019-12-10

**Authors:** Leandro R. Jones, Julieta M. Manrique, Noelia M. Uyua, Brian A. Whitton

**Affiliations:** 1 Laboratorio de Virología y Genética Molecular, Facultad de Ciencias Naturales y Ciencias de la Salud, Universidad Nacional de la Patagonia San Juan Bosco, 9 de Julio y Belgrano s/n, (9100) Trelew, Chubut Argentina; 2 Consejo Nacional de Investigaciones Científicas y Técnicas (CONICET), Buenos Aires, Argentina; 3 Durham University, Department of Biosciences, Durham, DH1 3LE UK; 4 Present Address: Instituto de Investigación de Hidrobiología, Facultad de Ciencias Naturales y Ciencias de la Salud, Universidad Nacional de la Patagonia San Juan Bosco, Gales 48, (9100) Trelew, Chubut Argentina

**Keywords:** Invasive species, Phylogenetics

## Abstract

The diatom *Didymosphenia geminata* has gained notoriety due to the massive growths which have occurred in recent decades in temperate regions. Different explanations have been proposed for this phenomenon, including the emergence of new invasive strains, human dispersion and climate change. Despite the fact in Argentina nuisance growths began in about 2010, historical records suggest that the alga was already present before that date. In addition, preliminary genetic data revealed too high a diversity to be explained by a recent invasion. Here, we estimate the divergence times of strains from southern Argentina. We integrate new genetic data and secondary, fossil and geological calibrations into a Penalized Likelihood model used to infer 18,630 plausible chronograms. These indicate that radiation of the lineages in Argentina began during or before the Pleistocene, which is hard to reconcile with the hypothesis that a new variant is responsible for the local mass growths. Instead, this suggests that important features of present distribution could be the result of multiple recent colonizations or the expansion of formerly rare populations. The text explains how these two possibilities are compatible with the hypothesis that recent nuisance blooms may be a consequence of climate change.

## Introduction

The colonial diatom *Didymosphenia geminata* (Lyngbye) Schmidt has been abundant in some rivers of the north temperate zone for many years. However, there are very few records before the last 30 years for other regions of the World^1,2^. In 1989 growths suddenly developed in rivers of the central region of Vancouver Island in Canada. Since then, many rivers from temperate regions worldwide have been studied carefully enough to be certain that there have also been major increases. Mass growths consist of epiphytic and epilithic accretions of thick mats mainly composed of branched stalks made of a matrix of polysaccharides, uronic acids and proteins^3,4^. The stalk mass greatly exceeds material inside the cell. *D. geminata* colonies can grow together with other benthic diatoms. In addition, organic debris and small organisms are often entangled in the mats. Mass growths are sometimes sufficient to cover not only the bed of a river, but also its vegetation. Although the alga is not considered harmful for human health, it is widely accepted that it has the potential of altering an ecosystem and producing negative impacts on human activities^5,6^. This has generated much concern and prompted control programs in some countries.

There is still no precise explanation for the behavior of the alga in recent decades. The temporal co-occurrence of mass growths along with increased sport fishing activity during the early Vancouver Island blooms^7^ fueled the hypothesis that the overgrowths could be due to dispersion by recreational fishermen. In addition, the facts that *D. geminata* rapidly invaded many New Zealand rivers and has been reported to be introduced species there^8,9^ have suggested that the nuisance growths elsewhere also were due to colonization of ecosystems in which the alga was formerly absent. That the overgrowths could be a consequence of the emergence of a new variant with increased growth and invasion capabilities has also been considered. These hypotheses were rapidly and widely accepted, strongly influencing management actions. Ecological, histochemical and experimental studies have reported a relationship between dissolved reactive phosphorus and *D. geminata* mass growths, which has led some authors to propose a link between the abnormal overgrowths and altered phosphate release regimes^7,10,11^. However, Ellwood and Whitton^12^ and Whitton *et al*.^2^ suggested it was increased concentrations of organic, rather than dissolved reactive phosphate, resulting from climate change which were responsible. Later on, parameters other than phosphorous (but that also can be affected by climate change), such as pH, hydrology (discharge and water movement) and irradiation, also have been suggested as possible bloom triggers, based on statistical correlations^6,13–15^.

Since 2010, the presence of *D. geminata* has been reported in several rivers and lakes distributed from about parallel −35° to Tierra del Fuego island at the Southern extreme of South America (reviewed in reference^16^). The hypothesis of a new invasive species has had quite a lot of acceptance. However, there is a record of the alga in the Bosque Andino Patagónico ecoregion that date back to the 1960s^17^, that is more than 40 years before the nuisance overgrowths wave. Asprey *et al*.^17^ reported the presence of *D. geminata* only at two out of eight sites surveyed and rare compared to other algae. The collected material was seen by one of the authors of the present study (BAW), who could confirm it corresponded to *D. geminata*. However, the sampling procedure used by J. F. Asprey in the field was designed for large plankton^17^; the presence of the alga in its usual environment, attached to river bed and submerged vegetation, was not investigated. There is only one further mention of *D. geminata* before 2010, for the Mejillones commune in Chile^18^. Neither the exact collection date of the Mejillones material nor the corresponding sampling method are provided in reference^18^. However, based on other data mentioned therein, the specimens could have been collected sometime between the end of XIX century and the first half of century XX. Thus, benthic data before 2010 are insufficient to be certain that *D. geminata* was not already present in the region before the recent overgrowths. Moreover, molecular studies have uncovered an interesting and apparently complex scenario. Chloroplast intergenic sequences from New Zealand are much homogeneous than sequences from elsewhere, which has been interpreted as evidence that the species is new in New Zealand^9^. Conversely, preliminary *18S* data from Argentina revealed a diversity too high to be explained by a recent introduction, especially if we consider that intergenic regions are expected to diverge faster than ribosomal genes^16^. This, together with the existence of historical records, suggests the possibility that *D. geminata* could be a formerly rare native (or anciently introduced) species.

In this work, we set out to contrast the alternative explanations for the nuisance growths in Argentina using genetic data. The incipience of molecular studies results in difficulties to generate sequence data because it requires using primers designed based on the few available *D. geminata* sequences, or primers targeting a wide range of taxa. The later procedure is not a straightforward solution, because other microorganisms and organic debris are usually entangled into *D. geminata* mats, the material usually chosen for sampling, which results in co-amplification of sequences from other taxa when using nonspecific primers. The possible solutions include cloning the sequences amplified by nonspecific primers (followed by selection of the clones harboring *D. geminata* sequences) or physically isolating cells prior to PCR amplification, which is the strategy used in the present study. Furthermore, *D. geminata* mats, and probably cells, are recalcitrant to DNA extraction and downstream molecular applications, hence some samples cannot be amplified despite the use of cell isolation and/or optimized protocols^19^. Here, we optimized the conditions to generate nuclear ribosomal DNA (*rDNA*; *18S*, *ITS1*, *5.8S*, *ITS2* and *28S loci*), mitochondrial cytochrome c-oxidase subunit 1 (*COX-1*) and plastid *rbcl* sequences. We then inferred the time that should have elapsed to generate the local diversity. We reasoned that, if the Argentine strains represent the descendants of a new, highly invasive variant, then the time to their most recent common ancestor (*tMRCA*) should be small, in the order of some tens of years. Otherwise, the *tMRCA* should be of a few My, based on the oldest fossils known for the species and related taxa worldwide.

## Results

### Molecular data generation

We obtained no, poor and/or non-specific amplification products despite previously published PCR conditions being implemented using either whole mat material (*WBS-DNA*) or isolated cells (*IC-DNA*) as template (not shown). This could, however, be improved by assessing several primer combinations and nesting schemes and carefully tuning the corresponding annealing temperatures. For each primer combination evaluated (Tables 1 and 2), the annealing temperature was optimized by a gradient PCR (50 to 65 °C in steps of ~1.5 °C) using *WBS-DNA* as template. Once optimal temperatures were determined, these were re-assessed using *IC-DNA* as template. The results of these experiments, including PCR performance and optimized temperatures, are outlined in Table 2. Out of 10 primer combinations evaluated, 8 were able to generate amplicons suitable for direct sequencing after PCR optimization (Table 2). All the samples could be amplified with primer pairs *EUK528f*-*etts3rev* and *eits2dir*-*etts3rev* (Experiment E in Table 2), but the obtained sequences corresponded to contaminant, environmental DNA. For one of the samples from the Futaleufú River (*FTa*), the amplified *28S* sequences did not correspond to *D. geminata* and the *rbcl* PCR produced negative results. No data could be generated for five samples from four different rivers, because no DNA could be amplified (samples *QQ*, *QM* and *TR* and *18S*, *ITS1-2*, *5.8S* and *rbcl* loci from samples *DVa* and *DVb*) or because the amplified sequences didn’t correspond to *D. geminata* (*28S* amplicons from samples *DVa* and *DVb*).

**Table 1:** Oligonucleotides used in PCR assays.

Primer	Sequence (5´ - 3´)^a^	Reference
*GazF1*	GTAGGTGAACCTGCGGAAGGA	^52^
*GazR2*	GGATGACCAAARAACCAAAA	^52^
*COI-F*	ATGATHGGDGCDCCWGAYATG	^23^
*COI-R*	CCWCCHCCHGCDGGRTC	^23^
*DPrbcL1*	AAGGAGAAATHAATGTCT	^54^
*DPrbcL7*	AARCAACCTTGTGTAAGTCTC	^54^
*rbcL-F*	ATGTCTCAATCTGTAWCAGAACGGACTC	^23^
*rbcL-R*	TAARAAWCKYTCTCTCCAACGCA	^23^
*D602F*	GTTGGATTTGTGATGGAATTTGAA	^21,51^
*D1670R*	CACCAGTAAAGGCATTAGCTG	^21,51^
*UNI17F*	ACCTGGTTGATCCTGCCAG	^55^
*UNI1534R*	TGATCCTTCYGCAGGTTCAC	^55^
*EUK528f*	CCGCGGTAATTCCAGCTC	^53^
*eits2dir*	GTAGGTGAACCTGCGGAAGGA	^53^
*etts3rev*	GGGGAATCCTTGTTAGTTTC	^53^
*ITS-F*	CSMACAACGATGAAGRRCRCAGC	^23^
*ITS-R*	TCCCDSTTCRBTCGCCVTTACT	^23^
*D1R-F*	ACCCGCTGAATTTAAGCATA	^56^
*D3B-R*	TCGGAGGGAACCAGCTACTA	^56^
*D2C-R*	CCTTGGTCCGTGTTTCAAGA	^56^

^a^Non-DNA characters correspond to IUPAC codes.

**Table 2:** PCR optimization outline.

Experiment	Target^a^	PCR Round	Forward	Reverse	WBS-DNA^b^	IC-DNA^b^	Annealing^c^
A	COX-1	1	*GazF1*	*GazR2*	+/−	ND	50.0
		2	*COI-F*	*COX-R*	+	+	59.9
B	rbcL	1	*DPrbcL1*	*DPrbcL7*	+/N	+	52.9
		2	*rbcL-F*	*rbcL-R*	+	+	55.0
C	18S	1	*D602F*	*D1670R*	+/N	+	52.3
D	ITS1-5.8S-ITS2	1	*eits2dir*	*etts3rev*	+	ND	52.4
		2	*ITS-F*	*ITS-R*	N	N	63.8
E	ITS1-5.8S-ITS2	1	*EUK528f*	*etts3rev*	+/N	+	59.1
		2	*eits2dir*	*etts3rev*	+	+	55.5
F	ITS1-5.8S-ITS2	1	*602F*	*etts3rev*	+/N	+	52.4
		2	*eits2dir*	*etts3rev*	+	+	55.5
G	ITS1-5.8S-ITS2	1	*602F*	*etts3rev*	+/N	+	52.4
		2	*ITS-F*	*etts3rev*	+	+	55.5
H	ITS1-5.8S-ITS2	1	*602F*	*ITS-R*	+/N	+	52.4
		2	*eits2dir*	*ITS-R*	+	+	55.5
I	ITS1-5.8S-ITS2	1	*602F*	*ITS-R*	+/N	+	52.4
		2	*ITS-F*	*ITS-R*	+	+	55.5
J	28S	1	*D1R-F*	*D3B-R*	+/N	ND	63.8
		2	*D1R-F*	*D2C-R*^*b*^	+/N	+	52.9

^a^Coding/transcribed sequence.

^b^PCR performance using mat (*WBS*) or isolated cells (*IC*) DNA as template. *ND* not detectable; +*/−* presence of faint band of the expected size; *+* presence of a good quality band of the expected size; *D/+* some samples negative; *N* nonspecific amplification, +/N presence of a good quality band of the expected size accompanied by nonspecific bands.

^c^Optimized annealing (this study); °C.

### Phylogenetic inference

As a first step in the dating analyses, we placed the Argentine sequences phylogenetically in the context of other diatom groups. This was done to ensure including a representative sample of the diatom diversity for estimating our molecular clock rates. Overall, we could compile *18S*, *28S* and *rbcl* sequences from 76 diatom species encompassing the major groups^20–33^. There were too few *COX-1* sequences available for these taxa and the available *ITS* and *5.8S* sequences could not be aligned confidently, so these loci were not used in the dating analyses. Previous studies have reported conflicting phylogenetic signals between different genes in some diatoms, which has been attributed to phenomena like differential retention of paralogs, horizontal gene transfer, heteroplasmic variation and deep coalescence^24,31,34^. Thus, we identified all the species or specimens having discordant relationships between the markers studied. The criterion implemented was that phylogenetic assignments between all the loci after both Maximum Likelihood and Parsimony analyses were to be compatible. The specimens that did not satisfy this criterion were dismissed from posterior analyses. The selected sequences are listed in Table 3. These sequences were aligned together with the Argentine *D. geminata* sequences and submitted to the evolutionary analyses described below.

**Table 3:** Diatom and outgroup sequences used in evolutionary analyses.

Species	*18S*	*28S*	*rbcl*	Reference
*Anomoeoneis sculpta*	KJ011611.1	KJ011556.1	KJ011794.1	^30^
*Cymbella aspera*	KJ011617.1	KJ011560.1	KJ011799.1	^30^
*Cymbella helvetica*	KJ011621.1	KJ011565.1	KJ011804.1	^30^
*Cymbella mexicana*	KJ011624.1	KJ011568.1	KJ011807.1	^30^
*Cymbella proxima*	KJ011625.1	KJ011569.1	KJ011808.1	^30^
*Cymbella tumida*	KJ011629.1	KJ011573.1	KJ011812.1	^30^
*Cymbopleura inaequalis*	KJ011631.1	KJ011575.1	KJ011814.1	^30^
*Cymbopleura naviculiformis*	KJ011632.1	KJ011576.1	KJ011815.1	^30^
*Didymosphenia dentata*	KJ011635.1	KJ011579.1	KJ011818.1	^30^
*Didymosphenia siberica*	KJ011637.1	KJ011581.1	KJ011820.1	^30^
*Gomphoneis minuta*	KJ011648.1	KJ011589.1	KJ011831.1	^30^
*Gomphonema clevei*	KC736623.1	JQ354596.1	KC736598.1	^26,27^
*Mayamaea atomus*	JN418600.1	JN418633.1	JN418670.1	^32^
*Pinnularia acuminata*	JN418597.1	JN418630.1	JN418667.1	^32^
*Pinnularia altiplanensis*	JN418573	JN418606	JN418643	^32^
*Pinnularia grunowii*	JN418588	JN418621	JN418658	^32^
*Pinnularia microstauron*	JN418568	JN418601.1	JN418638	^32^
*Pinnularia nodosa*	JN418587	JN418620	JN418657	^32^
*Pinnularia sp*.	JN418581.1	JN418614	JN418651	^32^
*Pinnularia subcommutata*	JN418584	JN418617	JN418654	^32^
*Pinnularia viridiforme*	JN418589.1	JN418622.1	JN418659.1	^32^
*Pinnularia viridiformis*	JN418574	JN418607	JN418644	^32^
*Sellaphora blackfordensis*	JN418599.1	JN418632.1	JN418669.1	^32^
*Cocconeis_stauroneiformis*	AB430614.1	AB430654.1	AB430694.1	^29^
*Cyclophora_tenuis*	AJ535142.1	AB430634.1	AB430673.1	^29^
*Fragilaria bidens*	AB430599.1	AB430636.1	AB430676.1	^29^
*Pteroncola inane*	AB430607.1	AB430647.1	AB430687.1	^29^
*Nanofrustulum shiloi*	AM746971.1	AB430640.1	AB430680.1	^29^
*Opephora sp*.	AB430604.1	AB430643.1	AB430683.1	^29^
*Plagiostriata goreensis*	AB430605.1	AB430644.1	AB430684.1	^29^
*Pseudostaurosira brevistriata*	AB430608.1	AB430648.1	AB430688.1	^29^
*Licmophora paradoxa*	AB430601.1	AB430639.1	AB430679.1	^29^
*Tabularia laevis*	AB430610.1	AB430650.1	AB430690.1	^29^
*Pseudohimantidium pacificum*	AB430606.1	AB430645.1	AB430685.1	^29^
*Grammatophora marina*	AB430600.1	AB430637.1	AB430677.1	^29^
*Hyalosira delicatula*	AF525654.1	AB430638.1	AB430678.1	^29^
*Hyalosira tropicalis*	B430612.1	AB430652.1	AB430692.1	^29^
*Rhabdonema minutum*	AB430603.1	AB430642.1	AB430682.1	^29^
*Diatoma moniliforme*	AB430597.1	AB430635.1	AB430674.1	^29^
*Thalassiothrix longissima*	AB430611.1	AB430651.1	AB430691.1	^29^
*Dimeregramma minor*	AB430598.1	AB425083.1	AB430675.1	^29^
*Rhaphoneis sp*.	AB430602.1	AB430641.1	AB430681.1	^29^
*Aulacoseira granulata*	AB430586.1	AB430619.1	AB430659.1	^29^
*Stephanopyxis turris*	KJ671710.1	AB430623.1	KJ671818.1	^23,29^
*Hyalodiscus scoticus*	AB430587.1	AB430620.1	AB430660.1	^29^
*Eunotogramma laevis*	AB430593.1	AB430628.1	AB430668.1	^29^
*Cymatosira belgica*	X85387.1	AB430627.1	AB430667.1	^29^
*Odontella sinensis*	Y10570.1	AB430630.1	Z67753.1	^28,29^
*Cyclotella meneghiniana*	AB430591.1	AB430625.1	AB430665.1	^29^
*Stephanodiscus sp*.	AB430594.1	AB430631.1	AB430670.1	^29^
*Skeletonema tropicum*	KJ671709.1	AB572824.1	KJ671817.1	^23,33^
*Thalassiosira nordenskioeldii*	KJ671714.1	HM991680.1	KJ671822.1	^23^
*Dictyota dichotoma*	AF350227.1	AF331152.1	AY422654.1	^22,29^
*Heterosigma akashiwo*	LC214052.1	AF409124.1	AB176660.1	^20^

**Figure 1:**
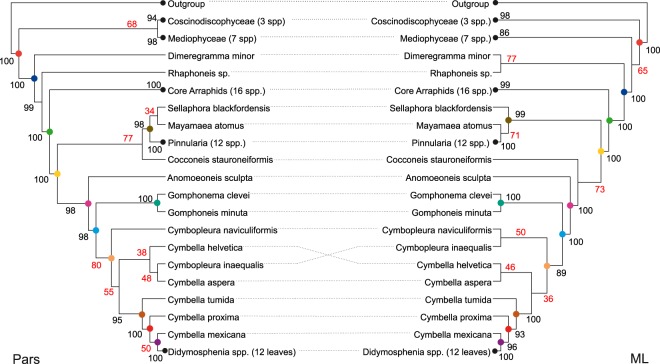
Maximum Likelihood (*ML*) and Parsimony (*Pars*) phylogenies of the sequences used in dating analyses. Black dots indicate clades that were collapsed for enhanced display (numbers of accrued terminals are given in parentheses in tip names). Colored dots on internal nodes indicate the clades that were recovered by both Parsimony and Maximum Likelihood analyses. Numbers close to branches correspond to bootstrap supports. Numbers in red indicate no (<50) or low (<80) support.

**Figure 2:**
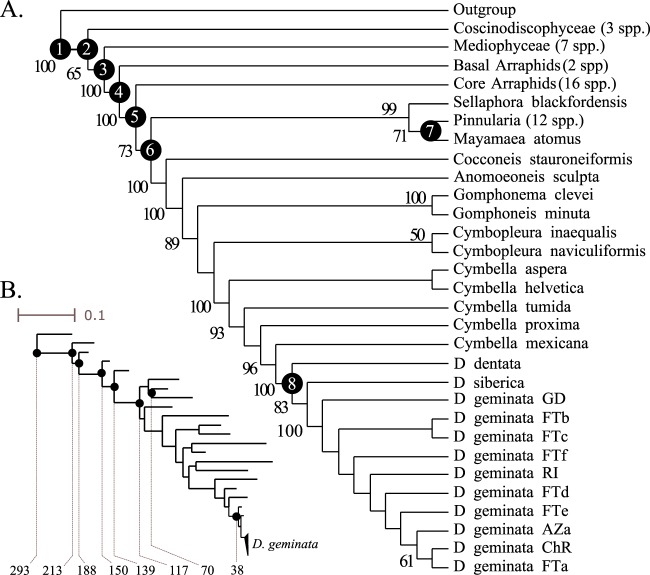
Calibration points implemented in dating analyses. A cladogram (**A**) and a phylogram (**B**) are displayed, corresponding to the topology obtained by Maximum Likelihood. Closed circles on nodes correspond to calibration points (white numbers on circles in panel A coincide with node numbers in Table 4). All the calibrated nodes but the node labeled 3 were shared with the corresponding Parsimony phylogeny (please see Fig. 1 and main text for detailed comparisons). Some clades were collapsed for enhanced display (numbers of accrued sequences are given in parenthesis in the corresponding terminal names). Numbers close to branches correspond to bootstrap supports > 50. Numbers below the phylogram correspond to mid-ranges between the minimum and maximum bounds implemented in the clock model (see also Table 4). Each *D. geminata* terminal is representative of 50 individual cells.

The aligned data presented 3754 positions with 1872 patterns of which 1341 were parsimony informative. Phylogenetic analyses recovered the major diatom groups with good branch supports (Fig. 1). The few nodes that were inconsistent between the Maximum Likelihood and Parsimony topologies presented low (<80) or no (<50) bootstrap supports. Furthermore, most alternative resolutions of such nodes were coincident with the most frequent resolution in the alternative method. For example, Mediophycea*e* was the sister clade of Bacillariophyceae (*i.e*. same as in *ML* tree) in 30 parsimony bootstrap trees and it was the sister clade of Coscinodiscophyceae (*i.e*. same as in Parsimony analysis) in 30 *ML* bootstrap topologies. Likewise, *Cocconeis stauroneiformis* was placed as the sister clade of Naviculales in 23 *ML* bootstrap trees and displayed the same position as in the *ML* bootstrap tree among 18 Parsimony bootstrap trees. *Cymbella mexicana*, *C. proxima* and *Didymosphenia* were always recovered as a monophyletic group. Nonetheless, the positioning of *C. mexicana* was erratic among the Parsimony bootstrap trees, where it was recovered either as the sister species of *Didymosphenia* (Fig. 1), the sister of *C. proxima* (17 trees), forming a trichotomy with *Didymosphenia* and *C. proxima* (8 trees) or as the sister species of (*Didymosphenia* + *C. proxima*) (25 trees). Uncertainty was considerable regarding the phylogenetic placement of other Cymbellaceae (Fig. 1). *Didymosphenia* and *D. geminata* were strongly supported in both analyses. *D. siberica* was the sister of *D. geminata* in 75 Parsimony Bootstrap trees and in 83 *ML* ones (Fig. 2). The most frequent alternative resolutions were *D. dentata* and *D. siberica* monophyletic (8 Parsimony and 8 *ML* bootstrap trees), the three species forming a trichotomy (8 Parsimony bootstrap trees) and *D. dentata* and *D. geminata* monophyletic (8 *ML* bootstrap trees). Despite *D. geminata* was well supported and the Parsimony analysis resulted in only 6 optimal topologies, the supports within the *D. geminata* clade were low, indicating that the data support different plausible phylogenies for our sequences. To take into account this uncertainty as well as the minor differences between the relationships of major diatom groups, we performed the dating analyses using the *ML* and Parsimony trees and the full sets of Parsimony and *ML* bootstrap trees (detailed below).

### Molecular clock parameters assessment

**Figure 3:**
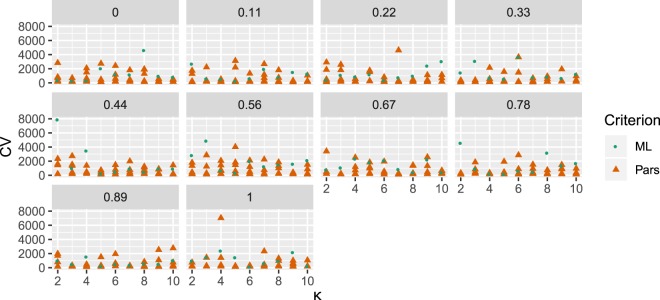
Cross-validation analyses. Each panel depicts the results obtained with a value of λ (0 to 1) combined with different numbers of rate categories (*k*; 2 to 10). *ML* Maximum Likelihood; *Pars* Parsimony.

**Figure 4:**
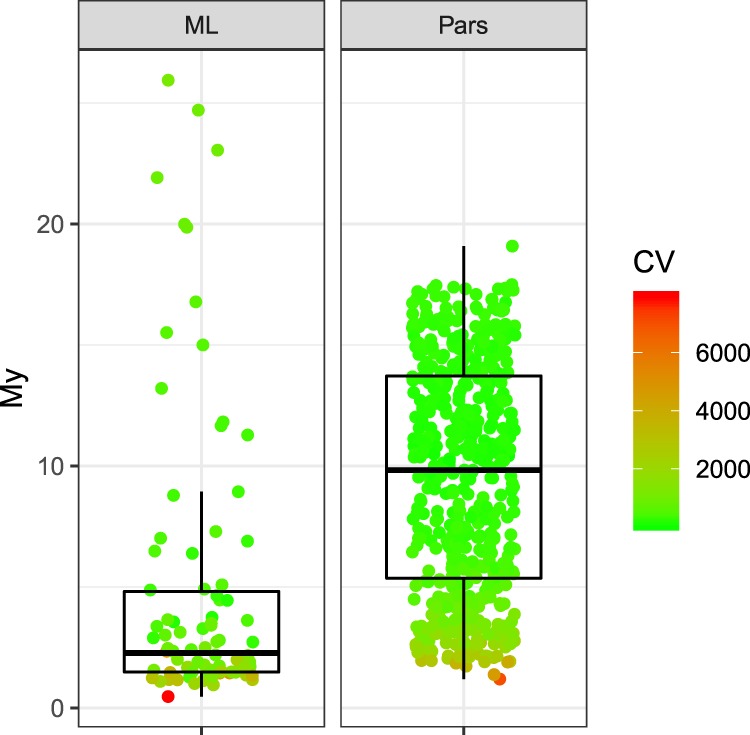
Time to the *MRCA* of the Argentine *D. geminata* strains inferred from optimum Maximum Likelihood (*ML*) and Parsimony (*Pars*) trees along all the λ and *k* combinations (Fig. 3). Points are colored according to the corresponding cross-validation scores (*CV*).

To assess the performance of different model parameter configurations, we implemented a cross-validation (*CV*) criterion (Materials and Methods). The smaller *CV* score (97.9) was obtained from one of our most parsimonious trees when it was analyzed using values of λ and *k* of 0.33 and 2, respectively. The *ML* tree resulted in the smaller *CV* when we set λ = 0 and *k* = 2. A few combinations of parameters resulted in very high *CV*s with some of our trees (Fig. 3). Nonetheless, these same configurations, in combination with other trees, produced average *CV*s. As instance, setting *k* = 4 and λ = 1 resulted in a very high *CV* when used with one of our most parsimonious trees. However, the same configuration produced relatively small *CV*s when used along the rest of our Parsimony and the *ML* trees. Furthermore, the majority of configurations and trees resulted in very similar *CV*s. In Fig. 4, where both the *tMRCAs* obtained from each reference tree and the corresponding *CV*s are shown, the majority of *tMRCAs* (points) are colored in intense green, meaning that the corresponding scores were small. In the same Figure, only a small fraction of the points is colored brown or red, which correspond to comparatively ‘bad’ (high) *CV*s. Moreover, it is easy to appreciate, by looking at Figs. 3 and 4 together, that these ‘bad’ *CV*s do not correspond to any combination of parameters. Thus, we integrated the results obtained under all the settings implemented.

### Divergence times estimation

**Figure 5:**
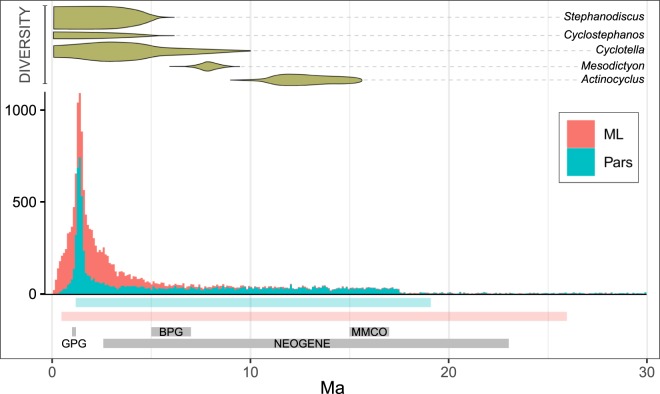
*D. geminata* chronology in Southern Argentina. The histogram summarizes the *tMRCAs* of the Argentine strains, inferred from 100 Maximum Likelihood (*ML*) and 100 Parsimony (*Pars*) bootstrap trees. Each tree was analyzed with 90 different λ and *k* combinations, giving a total of 18,000 chronograms. The color bars below the histogram represent the age ranges obtained using optimal *ML* and *Pars* trees (details in Fig. 4). The upper part of the figure shows the relative diversity of five diatom genera in the Neogene and Quaternary of USA; based on reference^36^. *GPG* Great Patagonian Glaciation (~1.17–1 Ma); *BPG* Beginning of Patagonian Glaciations (~7–5 Ma); *MMCO* Mid-Miocene Climatic Optimum (~15–17 Ma).

The *tMRCA*s obtained with the parameters that produced the lowest *CV*s were 10.41 My (Parsimony analysis) and 3.74 My (Maximum Likelihood analysis). The analyses of the optimal Maximum Likelihood tree along all the *k* and λ configurations (n = 90) produced *tMRCAs* of the Argentine *D. geminata* strains ranging from ~0.5 to ~26 My (Q1 = 1.48, Q3 = 4.81; Fig. 4), whereas the *tMRCAs* obtained using the six most parsimonious trees (n = 540) ranged from 1.19 to 19 My (Q1 = 5.36; Q3 = 13.72; Fig. 4). As mentioned above, to further weigh the effect of phylogenetic uncertainty, we also inferred chronograms from the bootstrap trees obtained by both *ML* and Parsimony. We used the full range of parameters’ combinations on each bootstrap tree, thus obtaining a total of 18,000 chronograms. The central 75% of the *tMRCAs* obtained from the *ML* and Parsimony chronograms combined fell in a time span running from 1.4 to 7.5 Ma. Individually, the *ML* bootstrap trees produced *tMRCAs* ranging from 0.06 to 74.75 My (Q1 = 1.17; Q3 = 3.09), whereas the corresponding Parsimony trees generated estimates ranging from 0.02 to 69.57 My (Q1 = 1.46; Q3 = 12). The whole analysis is summarized and compared to previous geological data and a fossil-based diatom biochronology in Fig. 5. Remarkably, many of the *tMRCAs* estimated here fell in a period of time roughly coinciding with the *Great Patagonian Glaciation* (*GPA*) between 1.17 and 1 Ma^35^. Moreover, the large majority of the obtained *tMRCAs* were coincident with a period of intense diatom turnover and diversification in the northern hemisphere^36^.

## Discussion

Here, we used genetic data to evaluate alternative explanations for the nuisance *D. geminata* overgrowths which have occurred in recent times in Argentina. Data from seven *loci* were generated, including nuclear (*18S*, *ITS-1*, *5.8S*, *ITS-2* and *28S*), mitochondrial (*COX-1*) and plastidic (*rbcl*) markers, and homologous sequences were searched among other groups of diatoms. The search resulted in a selection of sequences from 3 genes (*18S*, *28S*, and *rbcl*) that were well represented in the major diatom groups. These sequences were combined with geological, fossil and secondary calibrations to set a molecular clock that allowed us to infer a chronology for the strains present in Argentina. As discussed in detail below, the chronology suggests that the alga has recently become abundant or that several ancient *D. geminata* lineages have been recently introduced.

Generating *D. geminata* molecular data is difficult^19^. No *28S* and *COX-1* data have been reported before for this species. There is a single ribosomal *18S* sequence from the old world, obtained in the context of a broad study of other diatom groups^26^, and another from New Zealand^21^. The previous data from the American continent includes 28 molecular clone sequences of the *18S* gene from eastern rivers of the USA^25^, a single *18S* sequence from Colorado (USA) and 15 *18S* sequences from southern Argentina and Chile^16,24^. Sequence data have also been reported for the ribulose-1,5-biphosphate carboxylase/oxygenase large subunit (*rbcl*) gene of 9 samples from Chile^24^. The amplification of the *ITS1*, *5.8S* and *ITS2* loci resulted to be particularly challenging. All the primers combinations tested produced nonspecific or multiband amplifications except the ones including one didymo-specific primer in the first PCR rounds, which we attribute to an enhanced variability of these markers. On the other hand, the use of *D. geminata*-specific primers in the *18S* amplifications allowed us to generate good DNA amounts in a single PCR round using both *IC-DNA* and *WBS-DNA* as template. This can be helpful when molecular cloning or cell isolation cannot be afforded, as for example when analyzing large amounts of samples. Despite using optimized DNA extraction and PCR conditions, no data could be generated for five samples and two loci from a sixth. This might reflect the persistence of enzymatic inhibitors^19^. However, the amplification of environmental DNA in several experiments suggests that in some cases the absence of *D. geminata* amplicons might respond to sequence variability, which requires further study.

The chronology inferred for the strains from Argentina is summarized in Fig. 5. Overall, our results indicate that the common ancestor of the Argentine sequences probably lived during the Pleistocene or before. The younger *tMRCA* inferred from our 18,630 chronograms was 26,134 years and only about 10% (1911) of these chronograms supported *tMRCAs* shorter than 1 My. This makes it very unlikely that the nuisance growths from Argentina be caused by a novel variant of the species (in which case the actual *tMRCA* should be much smaller; maybe some tens of years). Taking into account the divergence times inferred in this work, in order for the ‘new variant’ hypothesis to be valid it would be necessary to assume that a mutant strain has emerged somewhere, and that this mutant was recently brought to the region and the mutation was transferred to ancient strains by sexual reproduction. We have observed populations with different average cell sizes, suggesting that cycles of cell size reduction and restitution may occur (unpublished results). However, to the best of our knowledge, there is no yet confirmation that size restitution is accompanied by sexual reproduction in *D. geminata*.

If it is assumed that the presence of *D. geminata* in South America is recent, a hypothesis based on our results is that the corresponding colonization was carried out by multiple ancient lineages. This idea (high propagule pressure) has been used to explain the high genetic diversity observed in two rivers in Maryland, USA^25^. A high propagule pressure could be a consequence, for example, of anglers spreading propagules consisting of many cells. As already explained, the concept that the recent nuisance behavior of the alga is associated with its dispersion along with fishing equipment is entrenched in some areas. However, previous studies indicate that *D. geminata* cells are easily killed by physical stress such as drying, salt and freezing^37^, which raises doubts about this explanation. The sensitivity of *D. geminata* cells to environmental stressors suggests that long-distance dispersion events are rare and probably achieved by very few cells. This concept is compatible with the genetic homogeneity observed in New Zealand^9^, where the species is likely a recent invader. A plausible way in which multiple *D. geminata* lineages could have arrived to South America is along with fish stocks brought to the region. P. J. Macchi and collaborators conducted a survey whereby they determined that between 1 and 8 foreign species were introduced into 28 basins between 1904 and 1965^38^. For example, more than 4 million exemplars of *Salvelinus fontinalis* were released in the Negro River basin in 1904 and about 3 million exemplars of *Onchorhynchus mykiss* in 1924^39^. However, it is not known if the conditions in man-made fish stocks permit *D. geminata* to survive.

Another possible explanation for the present results is that *D. geminata* may has been present in the region since ancient times but in very low abundance. There are previous fossil and extant taxa data that are compatible with this. Fossils of *Didymosphenia* spp. and *Cymbella mexicana*, the sister species of the genus *Didymosphenia* according to previous studies^30^ and our own results (Figs. 1 and 2), have been described in sedimentary rocks of the Miocene of North America and Japan^40,41^. This indicates that in that period there were already stem *Didymosphenia* species. In addition, the presence of *D. geminata* fossils has been reported in Pliocene sediments from China^42^. Thus, the species of the genus *Didymosphenia* likely have had plenty of time to disperse. By the other side, the cooling after the Mid-Miocene Climatic Optimum (*MMCO*) has allowed the establishment of temperate species in latitudes that formerly were too warm^43^. A nice example of this phenomenon is the diatoms biochronology studied by W. N. Krebs *et al*.^36^ (depicted in the upper panel of Fig. 5), which reflects the expansion and diversification of three diatom genera as a consequence of climate cooling in a region now corresponding to North America. The cooling after the *MMCO* also played a significant role in the configuration of the South American biota. Sérsic *et al*.^44^ and Hazzi *et al*.^45^ synthesized the available information for various groups of plants and animals. Hence, the species of the genus *Didymosphenia* have probably had the possibility of colonizing the land masses that now correspond to southern South America since the middle to late Neogene. This, together with the *tMRCA*s inferred here and the 20th century sporadic records, support the new hypothesis that *D. geminata* may has been present in the region since ancient times. The genetic diversity we observed could be consequence of the geoclimatic events occurred in the pre-Quaternary/Quaternary. For example, the Patagonian glacial cycles could have driven the establishment of various *D. geminata* lineages through phenomena like extinction/recolonization cycles (multiple introductions) and/or niche contraction/expansion (local diversification), as observed for other taxa from South America and elsewhere^44,46,47^. The divergence times inferred in this study are close to the divergence times of taxa believed to have been affected by Patagonian glaciations, which, in addition to the cooling and heating cycles, produced changes in the patterns of water courses and lakes, and the position of the continental divide. Our hypothesis predicts that *D. geminata* genotypes should have a more or less structured geographic distribution. Previous molecular studies revealed the existence of cryptic variation and a geographical structure between *Gomphonema parvulum* demes^48,49^, suggesting that it may also happen in *D. geminata*. Our hypothesis also predicts that there should be ancient *D. geminata* fossils in South American, but rare. Future research should aim to deepen genetic studies and investigate the fossil record, which will allow further insight on the possibility that *D. geminata* is an ancient, formerly rare species.

In summary, the results presented here are compatible with three possible scenarios: (i) that the recent overgrowths are due to the recent introduction of multiple lineages; (ii) that the species was already present and that for some reason it became more abundant; (iii) that a mutant lineage was recently introduced and dispersed new genes conferring an exacerbated invasiveness. The first two hypotheses, which are the ones we prefer for requiring less *ad-hoc* hypotheses, are compatible with the idea that an ecological factor may have been altered (or reached a threshold) recently, affecting (broadening?) the niche of the alga. Maybe a factor altered by climate change. Whitton *et al*.^2^ and Watson *et al*.^6^ reviewed the evidence suggesting that climate change has influenced some environmental factors and hence the competitive success of *D. geminata* elsewhere. If this is what happened in Argentina, we could be witnessing a conspicuous effect of climatic change and an example of its unpredictable consequences on worldwide distributed aquatic ecosystems.

## Materials and Methods

### Samples

*D. geminata* mats were collected at eight rivers distributed along about 1600 km in western Argentinean Patagonia and Tierra del Fuego. Sixteen samples (Table S1) were selected based on presence, abundance and integrity of cell content, complexity (*i.e*. proportion of didymo cells relative to debris and other microorganisms in the samples), river bed coverage (>50 m^2^), and geographic origin. The studied samples are from the Yelcho (samples *FTa*, *FTb*, *FTc*, *FTd*, *FTe*, *FTf* and *RI*; Futaleufú and Rivadavia Rivers), Puelo (samples *AZa*, *AZb* and QM; Azul and Quemquemtreu rivers), Chubut (sample CH; Chubut river), Santa Cruz (samples *DVa*, *DVb* and TR; De las Vueltas and Toro rivers), Valdivia (sample *QQ*; Quilaquina river) and Grande (sample *GD*; Grande River) basins. All the sampling sites are located in the Andean Patagonian Forest (Bosque Andino Patagónico, *BAP*) ecoregion except for Chubut River and Grande, which are close to the *BAP*/Patagonian Steppe ecotone. Details on sampling procedures and DNA extraction methods have been described elsewhere^16,19,50^.

### PCR amplification and sequencing

PCR amplifications were optimized using DNA templates (1 µl volume of purified DNA suspensions) obtained from both whole benthic samples (*i.e*. whole mat material; *WBS*) and didymo cells (50 per sample) isolated from the mats by mouth pipeting (*IC*). PCRs were made using oligonucleotides described in previous studies^21,23,51–56^, which are detailed in Table 1. All the reactions were carried out in a MyCycler Thermal Cycler (BioRad) applying programs consisting of an initial denaturation step at 95 °C for 1 min, followed by 30 cycles of 95 °C for 30s, an annealing step of 30s and an extension step at 72 °C for 1 min/Kb, and a final extension step of 72 °C for 1 min. PCR products were analyzed by electrophoresis in agarose gels (1.8% in 1X Tris-Acetate-EDTA buffer) stained with *Redgel* (Biotum) and visualization under UV light. A molecular weight marker, 1 Kb Plus DNA Ladder (Invitrogen), was included in parallel in each gel. For sequencing, three independent PCRs were purified using QIAquick PCR Purification Kit (QIAGEN) according to manufacturer instructions, and the obtained products were re-analyzed by electrophoresis, as mentioned before, but including a mass ruler (Low DNA Mass Ladder; Invitrogen) to quantify the DNA by densitometric analysis using the program Image J^57^. The purifications were also checked by photometric analysis using a Nano spectrophotometer Nano Vue plus (GE Health Science) and finally sequenced by Sanger chemistry using amplification oligos. Chromatogram data processing and sequence assemble were performed with the newer version of BioEdit^58^. Polymorphic sites (double peaks) were coded using IUPAC codes. The obtained sequences were deposited in GenBank under accession numbers KY007192-KY007220 and MK291483-MK291492 (Table S1).

### Sequence alignment

Sequences were aligned by the program *MAFFT* using the *linsi* iterative refinement method^59^. The obtained alignments were visually inspected using *Jalview* software^60^. For phylogenetic analyses, the different markers analyzed were aligned separately and then concatenated.

### Phylogenetic inference

Maximum Likelihood trees were inferred by the program RAxML next generation (*RAxML-ng*, DOI:10.5281/zenodo.593079) under evolutionary models inferred by *jModelTest 2*^61^ based on the Bayesian information criterion. The inferred models were very similar to each other. All included invariant sites and among-sites rate heterogeneity. The *rbcl* data was better explained by the GTR substitution scheme whereas for the rDNA sequences (*18S* and *28S*) the algorithm suggested one rate for A/C and C/G substitutions, a second one for A/T and G/T and 3 different rates for the remaining substitutions (the TIM3 model in *RAxML-ng*). Tree searches consisted in 20 initial Parsimony trees that were rearranged by tree bisection and reconnection (*TBR*). Parsimony analyses were made in *TNT* software^62^. Tree searches consisted in Wagner (*WAG*) initial trees that were subsequently rearranged by *TBR*. The number of *WAG* trees required to obtain a stable consensus tree (*sensu* Goloboff^63^ and references therein) was 100. Holding one tree during tree swapping was enough both to target optimal trees and obtain a stable consensus implementing 100 replications. Targeting all shortest trees (n = 6) required about 600 replications. Branch supports were calculated with the bootstrap routines implemented in *RAxML-ng* and *TNT*. Tree comparisons were performed with the *cophyloplot* function of the R package *Ape*^64^, *Dendroscope*^65^ and *ad-hoc* R scripts available from the authors on request.

### Penalized Likelihood (*PL*) dating

Penalized Likelihood analyses were performed with the *chronos* function from the R package *ape*^64^. We deemed the most realistic clock model for this dataset, the discrete one, in which different branches are characterized by discrete rate categories. This better reflects that our dataset included several diatom taxa (Table 3; details in Results). Implementing a discrete clock requires using a predefined number of rate categories (*k*) and a parameter, λ, which governs the magnitude of the penalty applied to rate changes across the phylogeny. The performance of different *k* and λ settings was explored by a cross-validation criterion. We implemented a one-by-one terminal removal approach as in reference^66^. We define a score (*CV*) based on the cross-validation implementation of *Ape*’s *chronopl* function, aiming to weigh the impact of parameters’ variation on *tMRCA* estimates. For each *k* and λ combination (varied from 2 to 10 in increments of 1 and 0 to 1 in increments of 1/9, respectively), the height of the *D. geminata* crown node (*OCH*, for Observed Crown Height) was obtained from the full tree (reference tree, *RT*) and from each of a set of *M* trees generated by deleting one *RT* terminal at a time. Then, we calculated a score for each combination of *k* and λ values by the equation:

$$CV=\mathop{\sum }\limits_{1}^{M}\frac{{(OCH-PCH)}^{2}}{OCH},$$

where *OCH* is the observed *D. geminata* crown node height, that is the height in the *RT*, and *PCH*s (Predictor Crown Heights) are the heights in the deleted trees.

### Tree calibration

*Didymosphenia* sp. fossils have been described from the Messinian period^41^, suggesting that *Didymosphenia* radiation started along the Neogene. Thus, we set the beginning of the Messinian as the minimum bound for the crown node of the genus. Previous studies^30^ and the analyses performed here (please see the Results) indicated that the most basal taxon inside the *Didymosphenia* clade is *D. dentata*, which is one of the endemic species of the genus in Lake Baikal. The Baikal formation begun in the Late Cretaceous and it is estimated that the older Baikal taxa appeared about 70 Ma^67,68^, so we used this to set the maximum bound for the whole *Didymosphenia* clade. The age of the diatoms stem node was bounded between 190 and 396 Ma based on fossil evidence^69^ and previous molecular clock analyses involving an extensive sample of eukaryotic taxa^70^, respectively. The diatom crown node, the Bacillariophyceae crown node, the core araphids/raphids split and the raphid pennates crown minimum and maximum bounds were set according to references^71^ and^29^. The Mediophyceae stem node was assigned a minimum age of 110 My based on fossil records^72,73^. The corresponding maximum age was equalized to the minimum age of the diatom crown node. We also set minimum and maximum bounds for the *Pinnularia* stem node, based on fossil data and molecular clock analyses from^32^. The full set of calibration points implemented in the present study are summarized in Table 4 and Fig. 2.

**Table 4:** Time constrains (My) used in dating analyses.

Calibration point^a^	Min.^b^	Max.^b^	Evidence	Reference
Diatoms stem node (1)	—	397	Secondary	^70^
Diatoms stem node (1)	190	—	Fossil	^69^
Diatoms crown node (2)	160	267	Secondary	^29,71^
*Mediophyceae* stem node (3)	—	267		—
*Mediophyceae* stem node (3)	110	—	Fossil	^72,73^
*Bacillariophyceae* crown node (4)	96.5	204	Secondary	^29,71^
Core araphids/raphids split (5)	93.8	185	Secondary	^29,71^
Raphid pennates crown (6)	70	165	Secondary	^29,71^
*Pinnularia* stem node (7)	—	100	Secondary	^32^
*Pinnularia* stem node (7)	40	—	Fossil	^32^
*Didymosphenia* crown node (8)	—	70	Geological	^67,68^
*Didymosphenia* crown node (8)	7.3	—	Fossil	^41^

^a^Numbers in parentheses refer to nodes in Fig. 2.

^b^Million years.

## Supplementary information

Supplementary Information
