# Supplementary material for: Genetic analysis of the invasive alga Didymosphenia geminata in Southern Argentina: Evidence of a Pleistocene origin of local lineages
Source: Sci Rep. 2019 Dec 10;9:18706. doi: 10.1038/s41598-019-55155-1 (PMC6904681; doi:10.1038/s41598-019-55155-1)
Supplement: Supplementary file 1 — Supplementary Information [file 41598_2019_55155_MOESM1_ESM.pdf]

Genetic analysis of the invasive alga Didymosphenia geminata in Southern Argentina:
Evidence of a Pleistocene origin of local lineages

Leandro R. Jones[1,2,*], Julieta M. Manrique[1,2], Noelia M. Uyua[1,2,#] & Brian A. Whitton[3]

[1] Laboratorio de Virología y Genética Molecular, Argentina
Facultad de Ciencias Naturales y Ciencias de la Salud,
Universidad Nacional de la Patagonia San Juan Bosco;
9 de Julio y Belgrano s/n (9100), Trelew, Chubut,
[2] Consejo Nacional de Investigaciones Científicas y Técnicas (CONICET)
[3] Durham University, Department of Biosciences, Durham DH1 3LE, UK

[*] Corresponding author: CONICET - Laboratorio de Virología y Genética Molecular;
9 de Julio y Belgrano s/n, (9100), Trelew, Chubut, Argentina.
E-mail: lrj000@gmail.com; ljones@conicet.gov.ar.
[#] Present Affiliation: Instituto de Investigación de Hidrobiología, Argentina
Facultad de Ciencias Naturales y Ciencias de la Salud,
Universidad Nacional de la Patagonia San Juan Bosco, Gales 48 (9100), Trelew, Chubut,

Supplementary Table 1. Details of sampling sites (Sampling site; Ecorregion), PCR amplification results (PCR; Template) and GenBank accession numbers (GI).

PCR:
ND: not detectable
+/-: presence of faint band of the expected size
+: presence of a good quality band of the expected size
N: nonspecific amplification
+/N: presence of a good quality band of the expected size accompanied by nonspecific bands.
eDNA: Environmental DNA (cross PCR reaction; see main text)
D.g. : *D. geminata* sequence not uploaded to GenBank

Sampling site:
Hyperlink locator (copy and paste in browser)

Ecorregion:
BAP, Bosque Andino Patagónico
PS, Patagonia Steppe

Template:
WBS-DNA, DNA from whole mat material
IC-DNA, DNA from isolated cells (50 cells per sample)

GI:
GenBank accession numbers

	**Code**	**FTa**	**FTb**	**FTb**	**FTc**	**FTd**	**FTe**	**FTf**	**GD**	**RI**	**DVa**	**DVb**	**TR**	**QQ**	**AZa**	**Azb**	**QM**	**ChR**
	**Basin**	Yelcho	Yelcho		Yelcho	Yelcho	Yelcho	Yelcho	Grande	Yelcho	Santa Cruz	Santa Cruz	Santa Cruz	Valdivia	Puelo	Puelo	Puelo	Chubut
	**River**	Futaleufú	Futaleufú		Futaleufú	Futaleufú	Futaleufú	Futaleufú	Grande	Rivadavia	De las Vueltas	De las Vueltas	Toro	Quilaquina	Azul	Azul	Quemquemtre	Chubut
	**Sampling site**	http://maps.google.com/maps?q=-43.13694,-71.60442&t=k	http://maps.google.com/maps?q=-43.13694,-71.60442&t=k	http://maps.google.com/maps?q=-43.13694,-71.60442&t=k	http://maps.google.com/maps?q=-43.1671,-71.58847&t=k	http://maps.google.com/maps?q=-43.17303,-71.59444&t=k	http://maps.google.com/maps?q=-43.17208,-71.65081&t=k	http://maps.google.com/maps?q=-43.17767,-71.63106&t=k	http://maps.google.com/maps?q=-53.823773,-67.758718&t=k	http://maps.google.com/maps?q=-42.677854,-71.70151&t=k	http://maps.google.com/maps?q=-49.267929,-72.873619&t=k	http://maps.google.com/maps?q=-49.314667,-72.897507&t=k	http://maps.google.com/maps?q=-49.13204,-72.943251&t=k	http://maps.google.com/maps?q=-40.320004,-71.37077&t=k	http://maps.google.com/maps?q=-42.03708,-71.60347&t=k	http://maps.google.com/maps?q=-42.03708,-71.60347&t=k	http://maps.google.com/maps?q=-40.223149,-70.732967&t=k	http://maps.google.com/maps?q=-42.339462,-70.868903&t=k
	**Ecoregion**	BAP	BAP		BAP	BAP	BAP	BAP	Ecotone	BAP	BAP	BAP	BAP	BAP	BAP	BAP	BAP	Ecotone
	**Collection Date**	09/2011	04-2012	04-2012	04-2012	04-2012	04-2012	04-2012	01/2013	10/2013	03/2016	03/2016	03/2016	11/2013	10/2013	10-2013	02/3013	11/2015
**Locus/loci**	**Template**	IC-DNA	WBS-DNA	IC-DNA	IC-DNA	IC-DNA	IC-DNA	IC-DNA	IC-DNA	IC-DNA	IC-DNA	IC-DNA	IC-DNA	IC-DNA	IC-DNA	IC-DNA	IC-DNA	IC-DNA
28S																		
PCR (forward/revers; round)																		
	DIR-F/D3B-R; 1	ND	+/N	ND	+/-	ND	ND	+/-	ND	ND	ND	ND	ND	ND	ND	ND	ND	ND
	DIR-F/D2C-R; 2	+	+/N	+	+	+	+	+	+	+	+	+	ND	ND	+	ND	ND	+
		eDNA									eDNA	eDNA						
**GI**				MK291486	MK291487	MK291488	MK291489	MK291490	MK291491	MK291492					MK291483			MK291484
ITS-1/5.8S/ITS-2																		
PCR (forward/revers; round)																		
	eits2dir/etts3rev;1	ND	+	ND	ND	ND	ND	ND	ND	ND	ND	ND	ND	ND	ND	ND	ND	ND
	ITS-F/ITS-R;2	ND	N	N	N	ND	N	N	N	N	ND	ND	ND	ND	ND	ND	ND	ND
	EUK528f/etts3rev;1	+/N	+/N	+	+/N	+/N	+/N	+/N	+/N	+/N	+/N	+/N	+/N	+/N	+/N	+/N	+/N	+/N
	eits2dir/etts3rev;2	+	+	+	+	+	+	+	+	+	+	+	+	+	+	+	+	+
		eDNA		eDNA	eDNA	eDNA	eDNA	eDNA	eDNA	eDNA	eDNA	eDNA	eDNA	eDNA	eDNA	eDNA	eDNA	eDNA
	602F/etts3rev; 1	ND	+/N	+	ND	ND	ND	ND	ND	ND	ND	ND	ND	ND	ND	ND	ND	ND
	ITS-F/etts3rev;2	ND	+	+	+/-	+	+	+	+/-	+	ND	ND	ND	ND	+	ND	ND	+
	602F/etts3rev;1	ND	+/N	+	ND	+/N	+/-	ND	ND	ND	ND	ND	ND	ND	ND	ND	ND	ND
	eits2dir/etts3rev;2	+	+	+	+/-	+	+	+	+/-	+	ND	ND	ND	ND	+	ND	ND	+/-
	602F/ITS-R;1	ND	+/N	+	ND	+/N	ND	+/-	ND	ND	ND	ND	ND	ND	ND	ND	ND	ND
	ITS-F/ITS-R;2	+	+	+	+	+	+	ND	+	+	ND	ND	ND	ND	ND	ND	ND	+
	602F/ITS-R;1	ND	+/N	+	+/N	ND	ND	+/N	ND	ND	ND	ND	ND	ND	ND	ND	ND	+/N
	eits2dir/ITS-R;2	+	+	+	+	+	+	+	+	+	ND	ND	ND	ND	+	ND	ND	+
**GI**		MK291485	D.g.	MK291486	MK291487	MK291488	MK291489	MK291490	MK291491	MK291492					MK291483			MK291484
18S(*)																		
PCR (forward/revers; round)																		
	UNI17F-UNI1534R	+	+	+	+	+	+	+	+	+	+/-	ND	+/-	ND	+	ND	ND	+
	D602F-D1670R	+	+/N	+	+	+	+	+	+	+	ND	ND	ND	ND	+	ND	ND	+
		MK291485	(*)	MK291486	MK291487	MK291488	MK291489	MK291490	MK291491	MK291492					MK291483			MK291484
**GI**																		
COX-1																		
PCR (forward/revers; round)																		
	GazF1/GazR2;1	ND	+/-	ND	ND	ND	ND	ND	ND	ND	ND	ND	ND	ND	ND	ND	ND	ND
	COI-F/COX-R;2	+	+	+	+	+	+	+	+	+	ND	ND	ND	ND	+	ND	ND	+
**GI**		KY007204		KY007205	KY007206	KY007207	KY007208	KY007209	KY007210	KY007211					KY007202			KY007203
rbcL																		
PCR (forward/revers; round)																		
	DPrbcL1/DPrbcL7;1	ND	+/N	+	ND	ND	ND	+/N	+	+	ND	ND	ND	ND	+	ND	ND	+
	rbcL-F/rbcL-R;2	ND	+	+	+	+	+	+	+	+	ND	ND	ND	ND	+	ND	ND	+
**GI**				KY007214	KY007215	KY007216	KY007217	KY007218	KY007219	KY007220					KY007212			KY007213

(*) Preliminary 18S data, obtained from WBS-DNA, were presented in reference 15.
GI: KY007192, KY007193, KY007194, KY007195, KY007196, KY007197, KY007198, KY007199, KY007200 and KY007201
